# Removal of a Recurrent Calvarial Hemangioma Followed by Autologous Iliac Crest Bone Reconstruction: A Case-Based Experience

**DOI:** 10.3390/curroncol32100551

**Published:** 2025-09-30

**Authors:** Kostadin Gigov, Ivan Ginev, Dobromira Shopova

**Affiliations:** 1 Section of Plastic Reconstructive and Aesthetic Surgery and Thermal Trauma, Department of Propedeutics of Surgical Diseases, Faculty of Medicine, “St. George” University Hospital Plovdiv, Medical University of Plovdiv, “Peshtersko shausse Blvd 66”, 4002 Plovdiv, Bulgaria; ivan.ginev@phd.mu-plovdiv.bg; 2 Department of Prosthetic Dentistry, Faculty of Dental Medicine, Medical University of Plovdiv, 4000 Plovdiv, Bulgaria

**Keywords:** calvarial hemangioma, skull reconstruction, iliac crest graft, cranial surgery

## Abstract

Restoring the integrity of the calvaria due to diploic cavernous hemangioma removal possesses surgical complexity regarding the location of the tumor and the intricacy of the reconstruction method. We report a case of a 36-year-old male with a recurrent cavernous hemagioma, affecting the frontal bone. The patient had undergone surgical extirpation of the primary lesion six years ago in a different plastic surgery department with histological verification. He presented to our department with local recurrence at the same site. The lesion was completely resected, followed by calvarial reconstruction using autologous non-vascularized bone graft harvested from the iliac crest. Histology confirmed the recurrence of a benign cavernous hemangioma. Postoperative recovery was devoid of complications, and a follow-up CT scan after 6 months revealed no recurrence, with stable graft integration. A major insight of this manuscript includes the discussion of the benefits of using autologous bone in young patients instead of synthetic counterparts.

## Simple Summary

This case report presents a 36-year-old man with a recurrent cavernous hemangioma of the frontal bone. Six years after the initial surgery, the tumor reappeared at the same site. The lesion was fully removed, and the skull defect was reconstructed using a non-vascularized bone graft from the iliac crest. Histology confirmed a benign recurrence. The patient recovered without complications, and a 6-month follow-up CT scan showed no recurrence and stable graft integration. The report highlights the advantages of using autologous bone grafts, especially in younger patients, over synthetic materials.

## 1. Introduction

Hemangiomas of the skull are rare benign vascular tumors that account for less than 1% of all primary bone neoplasms. Among these, the calvaria, particularly the frontal bone, is the most favored site, although lesions may affect the entire cranial vault. It is typically a slow-growing condition and is often discovered incidentally during imaging for unrelated reasons, arising from the diploic space of the skull bones [1,2,3]. These tumors are comprised of proliferating blood vessels within the diploic space of the skull and are most commonly classified as either capillary, cavernous or venous hemangiomas based on histologic architecture. The cavernous subtype is the most frequently encountered in cranial cases. The etiology remains unclear but is thought to involve congenital malformations of vascular tissue or post-traumatic proliferations. Most patients are asymptomatic or present with a painless, slowly enlarging bony mass, often noted incidentally or during evaluation for cosmetic concerns [4,5,6]. Neurological deficits are rare unless the lesion exerts rapid and profound expansion and compresses vital structures. Radiologically, these tumors exhibit classic appearances, such as a “wagon wheel,” “sunburst,” or, in many cases, a spiculated mass-like pattern on CT, and high signal intensity on T2-weighted MRI images [7,8]. Surgical excision remains the treatment of choice, particularly in symptomatic patients or those with aesthetic disfigurement, rapid growth, or suspicion of malignancy. The standard surgical procedure involves performing a craniotomy with en bloc excision of the calvarial hemangioma to ensure complete removal of the lesion. If the resulting bone defect is significant or causes cosmetic concern, cranial reconstruction may be necessary. This can be achieved using various materials, such as titanium mesh, autologous bone grafts, or synthetic cranioplasty materials, depending on the size and location of the defect [9,10,11].

Complete resection with a margin of healthy bone is generally curative, and recurrence is uncommon. However, incomplete removal, particularly in cases where the lesion invades multiple calvarial layers or is near critical neurovascular structures, may predispose to local recurrence [12,13]. Reconstructive strategies following calvarial tumor resection must consider both functional protection of the brain and cosmetic restoration of the skull contour. While various materials, such as titanium meshes, polymethylmethacrylate (PMMA), and polyetheretherketone (PEEK) implants, are available, autologous bone grafting, particularly using the iliac crest, remains a highly effective and biocompatible option. Autologous grafts offer advantages in terms of osteointegration, lower infection and exposition risk, and long-term viability [14,15,16,17].

Embolization may be used as a pre-operative or palliative measure in the management of calvarial hemangiomas. It is particularly indicated for highly vascular lesions where there is a significant risk of intraoperative bleeding. In such cases, pre-surgical embolization can reduce vascularity and facilitate safer resection. Although not typically used as a definitive treatment, it plays an important supportive role in selected patients [18,19,20]. Radiotherapy is a rarely used treatment option for calvarial hemangiomas and is generally reserved for exceptional cases. It may be considered in patients with inoperable lesions or in cases of residual or recurrent tumor following surgical excision. While radiotherapy can help slow the growth of the tumor, it is not regarded as a first-line treatment due to potential risks to surrounding healthy tissue, particularly in the cranial region [21,22,23].

Although it is uncommon, congenital calvarial hemangioma should be included in the list of possible causes for slowly growing skull lesions, because it can lead to complications if it extends into the brain, and it may be treatable [24,25]. The differential diagnosis includes metastasis from prostate or lung cancer, myeloma in the form of solitary plasmocytomas, Langerhans histocytosis, Fibrous dysplasia, or Osteoma [26,27].

In this case report, we describe a 36-year-old male patient who presented with a recurrent frontal bone hemangioma six years after the initial surgical resection. He underwent successful tumor resection and calvarial reconstruction using autologous non-vascularized iliac crest bone with excellent cosmetic and clinical outcomes. This case underscores the importance of vigilant long-term follow-up and highlights the reconstructive potential of autologous bone in young patients with skull defects as a definitive treatment choice.

## 2. Case Report

A 36-year-old male patient presented to the clinic reporting a nearly 2-year history of expansive bulging underneath the skin affecting the frontal bone. The patient had undergone a surgical intervention 6 years ago in another plastic surgery department due to tumor formation in the exact same location, which, on histological examination, was confirmed to be a cavernous hemangioma.

**Figure 1:**
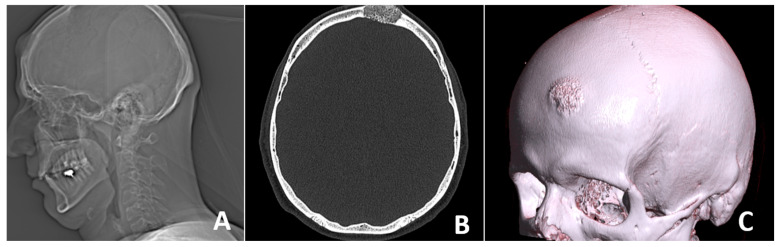
(**A**) X-ray and (**B**,**C**) CT scan prior to surgery reveal a bone lesion in the left frontal bone, involving the diploe, with a homogeneous structure, spiculated anterior contour, and local bone expansion.

X-ray and CT scan performed prior to surgery revealed the presence of a well-defined bone lesion located in the left frontal bone. The lesion involves the diploic space, demonstrating a homogeneous internal structure and a characteristic spiculated anterior contour. This radiographic feature is associated with a sunburst or radiating trabecular pattern, commonly seen in vascular bone lesions. Additionally, the lesion is accompanied by localized expansion of the surrounding bone, without evidence of cortical destruction or soft tissue invasion. The dimensions of the lesion are approximately 27 mm in length and 16 mm in width. Based on the imaging characteristics—including its location, internal architecture, and expansion pattern—the findings are highly suggestive of a calvarial hemangioma. Further surgical and histopathological evaluation was indicated to confirm the diagnosis and guide treatment (Figure 1).

**Figure 2:**
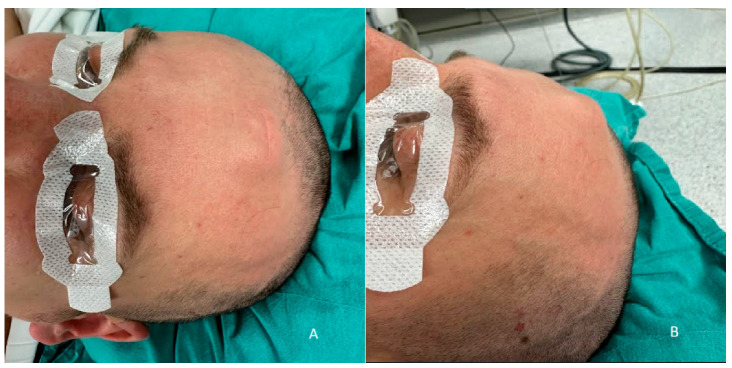
Lesion underneath the skin, with dense consistency and well-demarcated from surrounding tissues: (**A**) frontal view; (**B**) side view.

On physical examination, the lesion is presented as a dense mass with homogenous consistency, measuring nearly 3 cm in diameter. In the same location, there was a scar from the previous surgery, as shown in Figure 2**.**

**Figure 3:**
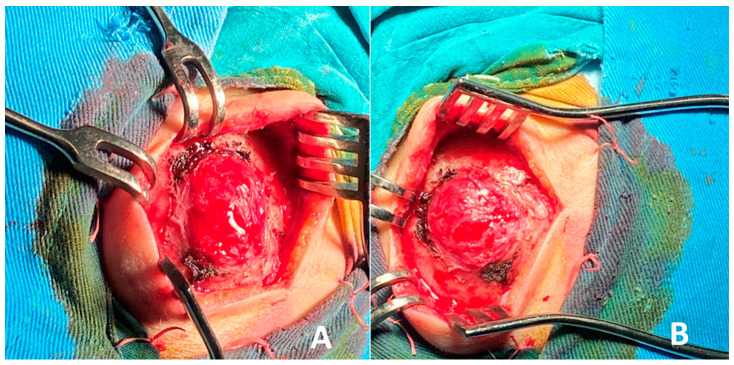
Vascular lesion, nearly 3 cm in diameter, affecting the diploe of the frontal bone: (**A**) central view; (**B**) side view.

A local recurrence of the hemangioma was suspected, and the surgical treatment plan included complete excision of the lesion followed by reconstruction with autologous iliac crest bone. The patient had no history of chronic disease and was prepared for elective surgery. The patient was placed under general intubation anesthesia in the supine position on the operating table. Ultrasound was used for marking the tumor borders. A straight incision through the old surgical scar in the frontal region was performed. The tumor process was visualized, with significant protrusion, causing an aesthetic defect, as shown in Figure 3.

**Figure 4:**
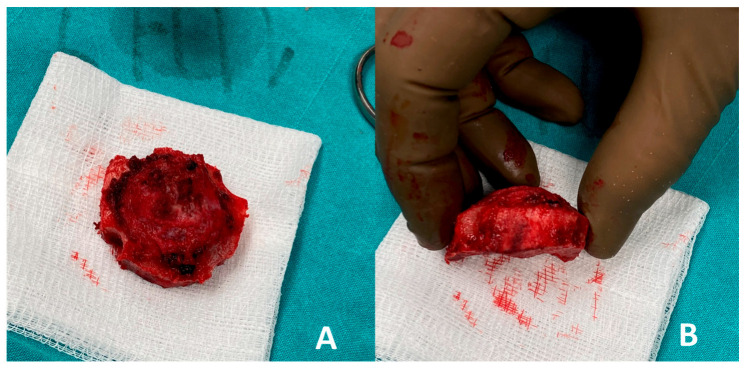
Complete removal of the tumor: (**A**) diameter; (**B**) thickness.

The frontal bone was exposed, and careful dissection of the overlying periosteum and surrounding soft tissues was performed to isolate the lesion. A craniotomy was then carried out by creating four burr holes strategically positioned around the lesion. These burr holes were subsequently connected using a bone saw to create a bone flap, allowing full access to the underlying lesion for en bloc excision. The technique ensured the preservation of adjacent structures and provided a controlled approach to remove the lesion with minimal trauma, as shown in Figure 4.

**Figure 5:**
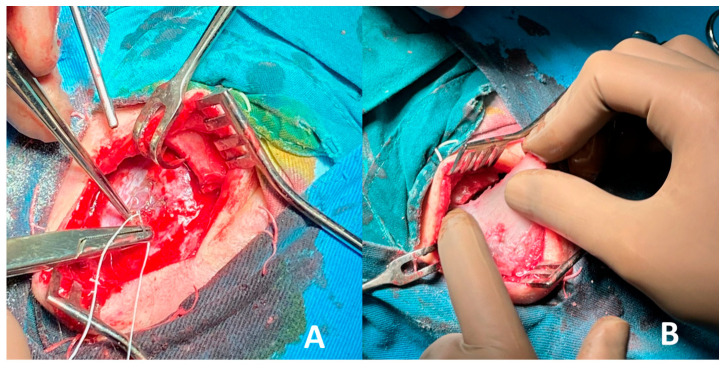
(**A**) The dura mater was precisely separated from the bone and the vascular lesion; (**B**) Iliac crest bone graft was harvested and molded to fit into the defect and mimic the normal contour of the frontal bone.

Using the created bone flap, the tumor formation was completely excised en bloc and was meticulously separated from the underlying dura mater, ensuring no dural injury occurred during the procedure. Following tumor removal, reconstruction of the cranial defect was performed using an autologous bone graft harvested from the right iliac crest. The graft was shaped to fit the defect precisely and securely fixed in place with sutures in the frontal area, restoring the anatomical contour and structural integrity of the skull, as demonstrated in Figure 5.

**Figure 6:**
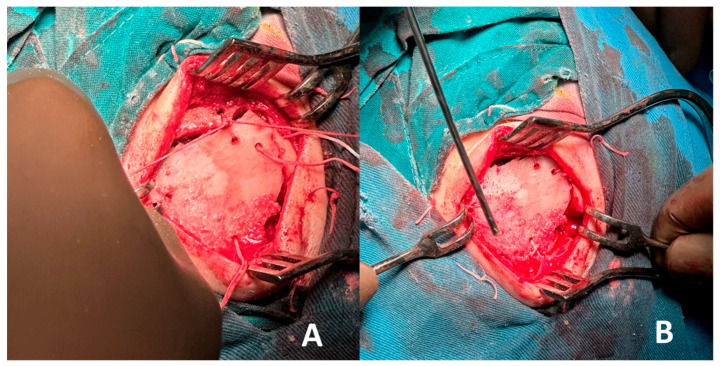
(**A**) Initial situation of graft positioning and sewing; (**B**) Final situation—Vicryl suture.

Good aesthetic correction of the frontal contour was achieved, with the reconstructed area closely resembling the native bone structure. Thorough hemostasis was performed to minimize the risk of postoperative bleeding. A surgical drain was placed at the donor site to prevent fluid accumulation. The layered closure of the wound was carried out meticulously to promote optimal healing. Small burr holes were carefully drilled into both the edges of the cranial defect and the autologous bone graft to facilitate secure fixation. The graft was then precisely positioned and anchored in place using absorbable Vicryl sutures, ensuring stability and proper alignment with the surrounding bone. This technique provided reliable fixation while maintaining a smooth external contour and supporting the healing process. Finally, sterile ointment dressings were applied to both the cranial and donor sites to protect the surgical areas and reduce the risk of infection, as shown in Figure 6.

**Figure 7:**
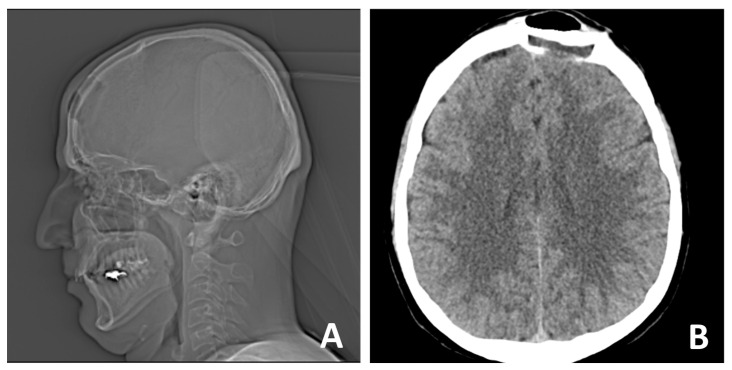
Control (**A**) X-ray and (**B**) CT was performed on the following day.

CT scan of the head—native (non-contrast) conditions was performed one day after surgery, showing normal density characteristics and preserved differentiation between white and gray matter in the brain structures. The ventricular system was symmetrical with preserved capacity. Normal position of the midline structures was observed. The posterior cranial fossa was intact, and the subarachnoid spaces were free. Bones—postoperative defect in the left frontal area. No complications were observed, as shown in Figure 7.

**Figure 8:**
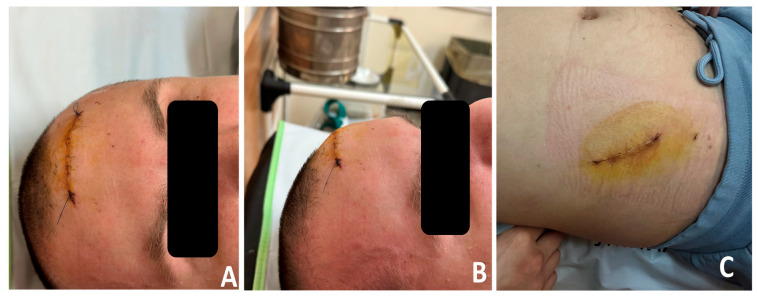
Follow-up situation after two weeks: (**A**) frontal view; (**B**) side view; (**C**) iliac zone.

Histopathological analysis of the excised tissue confirmed the diagnosis of cavernous diploic hemangioma, consistent with the preoperative imaging findings. Two weeks after surgery, the follow-up examination revealed a stable postoperative result with no evidence of skull protrusion or contour deformities. The surgical site showed good healing, and the aesthetic outcome remained satisfactory. No complications or morbidity were observed at the iliac crest donor site, and the patient reported no pain or discomfort during movement, as shown in Figure 8.

At the 6-month follow-up, the patient demonstrated a sustained long-term result with fully restored calvarial stability, ensuring adequate protection of the underlying brain structures. A follow-up CT scan of the head was performed again, showing normally presented brain structures and complete osteointegration of the iliac crest graft. The cosmetic outcome remained satisfactory, with no contour irregularities or visible deformities. No postoperative complications were observed, such as hematoma formation, infection, or extrusion of the bone graft. Overall, the patient maintained excellent functional and aesthetic results, confirming the success of the surgical intervention.

## 3. Results and Discussion

Frontal bone reconstruction following tumor resection presents a unique challenge in craniofacial surgery, requiring both aesthetic restoration and structural stability, necessary for brain protection. In our case, a 36-year-old male underwent frontal cranioplasty using an autologous iliac crest bone graft following en bloc resection of an osseous hemangioma involving the diploe of the frontal bone [7,8,19].

1. Autologous iliac crest bone grafts offer robust cortical and cancellous components suitable for reconstructing moderate to large cranial defects and remain the gold standard for calvarial reconstruction due to their osteoconductive, osteoinductive, and osteogenic properties. Despite potential donor-site morbidity, including pain, gait disturbance, hematoma, or sensory disturbance, the iliac crest remains a favored source due to its accessibility and structural quality [28,29,30]. In our case, the bone graft was successfully harvested and contoured to match the frontal defect and was secured with vicryl suture, achieving a stable reconstruction and satisfactory aesthetic outcome. Especially in the frontal region, calvarial grafts and iliac crest grafts yield excellent contour match and longevity.
2. Intraosseous cavernous hemangiomas of the skull often involve the diploic space. Although rare, cases treated with split-calvarial grafts after resection have shown good long-term outcomes, including stability and minimal recurrence over follow-up (~5 years) [29]. Such lesions affecting the diploe warrant en bloc removal with margins to prevent recurrences, followed by primary bone reconstruction [31]. Non-vascularized iliac crest bone grafts have also been used for frontal sinus obliteration, with minimal morbidity and no significant resorption at one and five-year follow-up. Vascularized iliac crest flaps (e.g., DCIA) have also been described in frontal cranioplasty, combining a muscle cuff and skin paddle for dead-space obliteration, offering rapid integration and sustained volume over time [29,32]. When local calvarium cannot be harvested or is inadequate, iliac crest grafts offer robust corticocancellous bone with sufficient volume and curvature to restore frontal contour. The iliac crest provides a broad surface suitable for large frontal defects and can be contoured to match convexity without multi-segment constructs [33,34].

Autologous grafts, especially iliac crest bone, integrate well via osteogenesis. Vascularized iliac bone grafts show reduced risk of resorption and earlier incorporation (callus formation within a week) compared to non-vascularized grafts. While vascularized iliac crest flaps are more complex, even non-vascularized autografts demonstrate low resorption and stable cosmetic results over the years in frontal reconstruction series [35,36]. Harvesting from the iliac crest can lead to donor-site issues: postoperative pain, possible pelvic instability, nerve injuries (lateral femoral cutaneous), hernia, or gait issues [37]. Pain correlates with graft size and typically subsides over time. Such risks should be balanced against the benefits, and meticulous technique (including posterior crest harvest and preservation of muscle and nerves) helps minimize complications [38]. Iliac crest graft must be contoured precisely to replicate normal forehead curvature and thickness. Scalp closure over a bone graft must be tension-free; in some cases, where there are larger defects, tissue expansion or the use of vascularized flaps may be needed, particularly in cases with large overlying scalp resections [7].

A similar principle of autologous reconstruction was employed by Bhardwaj et al. (2024) [15], who described a case of frontal bone reconstruction using a custom-designed titanium mesh combined with a costochondral graft harvested from the 10th rib. Their patient presented with a post-traumatic deformity of the frontal and naso-orbito-ethmoidal (NOE) region, necessitating both skeletal contouring and nasal dorsal support. In contrast to our case, their reconstruction incorporated alloplastic materials—specifically, a patient-specific titanium implant (PSI)—which allowed for precise anatomical restoration, particularly in cases with complex contours or secondary deformities [15]. While PSIs offer excellent biomechanical stability and aesthetic accuracy, they require time-intensive planning and production and carry the risks associated with foreign body implantation, including infection and extrusion [30,37]. In our case, primary reconstruction following tumor resection allowed for the use of autologous grafting alone, eliminating the need for alloplastic components. Moreover, iliac crest grafts offer greater volume and structural support compared to costochondral grafts, which are more suitable for smaller or cartilaginous reconstructions such as nasal framework [39].

Both approaches underscore the importance of individualized treatment planning based on defect characteristics, patient history, and resource availability. Our favorable outcome supports the viability of autologous iliac crest grafting for primary frontal bone reconstruction, especially when performed in a single-stage procedure. Long-term follow-up remains essential to monitor graft integration, contour stability, and donor-site morbidity [40].

A study conducted by Baudoin et al. (2021) [41] highlights vascularized split-iliac crest bone flaps as a reliable alternative to alloplastic implants or non-vascularized grafts in severely damaged, irradiated or recurrent tumor beds. The vascularized split-iliac crest bone flap, harvested with an intact arterial pedicle based on the deep circumflex iliac artery (DCIA), can be contoured to match the frontal bone defect and provide coverage even in cases where the sinus is affected [41]. Vascularized bone transfer facilitates excellent viability, structural support, and soft tissue healing, and establishes significance in patients with poor wound healing and compromised vascularity, where non-vascularized grafts have a higher potential for failure [33]. However, in young patients, who have high osteogenic and osteointegrative potential, without any chronic diseases or risk factors, the non-vascularized iliac crest bone graft, remains a safe and reliable option and reduces the potential complications, associated with vascular grafts, such as vascular anastomosis failure, increased donor-site morbidity, prolonged duration of surgery, hospital stay, and burden of care [25,42,43].

Alloplastic materials (PEEK, PMMA, and titanium mesh) can be used and are suitable for large defects, where sufficient autologous bone graft harvest is not possible. Their employment has increased significantly, especially after the incorporation of three-dimensional computer tomography. Nevertheless, they carry a higher infection risk, potential for rejection, and lack biologic remodeling—limitations that are especially significant in a vascular resection bed [44,45,46].

## 4. Conclusions

In young patients with frontal bone hemangioma involving the diploic space, reconstruction using autologous iliac crest bone represents a well-established and biologically compatible option. This approach offers reliable volume maintenance, good graft integration, and restoration of a natural cranial contour. The structural properties of the iliac crest graft make it particularly suitable for calvarial reconstruction, especially in defects requiring both functional and aesthetic restoration. However, surgeons must carefully consider potential donor site morbidity and ensure precise matching of graft thickness and curvature to the native bone to achieve optimal outcomes and minimize complications.

## Data Availability

Data are contained within the article.

## Abbreviations

The following abbreviations are used in this manuscript:

CT	Computed Tomography
DCIA	Deep Circumflex Iliac Artery
NOE	Naso-ethmoid-orbital
PSI	Patient Specific Implant
PMMA	poly-methyl-meta-acrylate
PEEK	polyetheretherketone
